# Cellular fatty acid synthase is required for late stages of HIV-1 replication

**DOI:** 10.1186/s12977-017-0368-z

**Published:** 2017-09-29

**Authors:** Manjusha M. Kulkarni, Annette N. Ratcliff, Menakshi Bhat, Yazan Alwarawrah, Philip Hughes, Jesus Arcos, David Loiselle, Jordi B. Torrelles, Nicholas T. Funderburg, Timothy A. Haystead, Jesse J. Kwiek

**Affiliations:** 1 Department of Microbial Infection and Immunity, Center for Microbial Interface Biology, The Ohio State University, Columbus, OH USA; 2 Department of Pharmacology and Cancer Biology, Duke University School of Medicine, C118 LSRC, Box 3813, Durham, NC 27710 USA; 3 Division of Medical Laboratory Science, School of Health and Rehabilitation Sciences, The Ohio State University, Columbus, OH USA; 4 Department of Microbiology, Center for Retrovirus Research, The Ohio State University, 476 Biological Sciences Building, 484 W. 12th Avenue, Columbus, OH 43210 USA; 5 Present Address: Promega Corporation, 2800 Woods Hollow Rd, Madison, WI 53711-5399 USA; 6 Present Address: Texas Biomedical Research Institute, San Antonio, Texas USA

**Keywords:** Human immunodeficiency virus type 1, Host-virus interaction, Fatty acid synthase, Antiviral pharmacology, Fasnall

## Abstract

**Background:**

Like all viruses, HIV-1 relies on host systems to replicate. The human purinome consists of approximately two thousand proteins that bind and use purines such as ATP, NADH, and NADPH. By virtue of their purine binding pockets, purinome proteins are highly druggable, and many existing drugs target purine-using enzymes. Leveraging a protein affinity media that uses the purine-binding pocket to capture the entire purinome, we sought to define purine-binding proteins regulated by HIV-1 infection.

**Results:**

Using purinome capture media, we observed that HIV-1 infection increases intracellular levels of fatty acid synthase (FASN), a NADPH-using enzyme critical to the synthesis of de novo fatty acids. siRNA mediated knockdown of FASN reduced HIV-1 particle production by 80%, and treatment of tissue culture cells or primary PBMCs with Fasnall, a newly described selective FASN inhibitor, reduced HIV-1 virion production by 90% (EC_50_ = 213 nM). Despite the requirement of FASN for nascent virion production, FASN activity was not required for intracellular Gag protein production, indicating that FASN dependent de novo fatty acid biosynthesis contributes to a late step of HIV-1 replication.

**Conclusions:**

Here we show that HIV-1 replication both increases FASN levels and requires host FASN activity. We also report that Fasnall, a novel FASN inhibitor that demonstrates anti-tumor activity in vivo, is a potent and efficacious antiviral, blocking HIV-1 replication in both tissue culture and primary cell models of HIV-1 replication. In adults, most fatty acids are obtained exogenously from the diet, thus making FASN a plausible candidate for pharmacological intervention. In conclusion, we hypothesize that FASN is a novel host dependency factor and that inhibition of FASN activity has the potential to be exploited as an antiretroviral strategy.

## Background

Viruses repurpose host cellular synthetic and metabolic pathways to produce progeny. Using large-scale CRISPR [1] or siRNA-based screens, several groups have identified host proteins required for replication of West Nile virus (WNV) [2], dengue virus (DENV) [3], hepatitis C virus (HCV) [4], influenza [5], and human immunodeficiency virus type-1 (HIV-1) [6–10]. These studies established the range of host proteins that modulate viral replication, and by extension, they also highlight the potential to develop antiviral drugs that target host proteins [7, 11]. Maraviroc, which targets host CCR5 molecules and blocks HIV-1 replication, is the prototypical host directed antiviral drug [11]. Recently, our laboratories and others have shown that inhibitors of the chaperone proteins Hsp70 and Hsp90 inhibit Chikungunya virus (CHIKV) and dengue fever virus (DENV) replication in cell and animal models [12–14]. Since host therapeutic targets evolve more slowly than viral therapeutic targets, antiviral therapy targeting host proteins would likely impose a high barrier to drug resistance. Further, if several viruses require the same host pathway, the potential exists to develop a pan-antiviral drug. The current challenge is to identify a host target that when inhibited, it limits viral replication while simultaneously not harming the host.

Here we report that HIV-1 infection increases host fatty acid synthase (FASN) levels, and a decrease in FASN activity attenuates HIV replication during a late stage of its replication cycle. We also report that Fasnall, a novel FASN inhibitor with anti-tumor activity [15] potently reduces HIV-1 production with minimal effects on cellular viability.

## Results

### Identification of purine-binding proteins regulated by HIV-1 infection

**Fig. 1:**
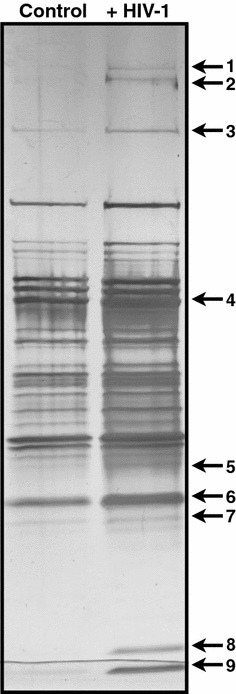
Purine-binding proteins regulated by HIV-1 infection. TZM-bl cells were HIV-infected and the TZM-bl purinome was captured 48-h post-infection. Proteins that remained bound to the resin after a high ionic wash were competed off the resin with 25 mM ATP, resolved by one-dimensional SDS-PAGE, visualized with silver stain, and identified with MALDI-TOF sequencing as the following: (1) Ubiquitin carboxyl terminal hydrolase (Q9Y4E8), (2) ATP-dependent RNA helicase DHX8 (Q14562), (3) FASN (P49327), (4) HSP90-beta (P08238), (5) GDP-L-fucose synthetase (Q13630), (6) L-lactate dehydrogenase (P07195) and pyridoxial kinase (O00764), (7) Argininosuccinate synthase (P00966), (8) Nucleoside diphosphate kinase-A (P15531) and, (9) Nucleoside diphosphate kinase–B (P22392)

We used an unbiased, functional chemoproteomic screen [16, 17] to define purine-binding proteins regulated by HIV-1 infection. HeLa-derived TZM-bl cells were infected with HIV-1, lysed 48 h post infection, and incubated with the purinome-capture resin. After extensive washing, bound proteins were competed off the purinome-capture resin with ATP. HIV-infection increased the recovery of several human proteins from the purinome-binding resin, including FASN, heat-shock protein 90 (HSP90), and others (Fig. 1). HSP90 is a validated cancer target and its role in HIV-1 replication has been reported elsewhere [18–20]. We focused on FASN (see band 3, Fig. 1), owing to its specialized, well-defined cellular function (de novo fatty acid synthesis), limited tissue expression [21], and previously reported association with flavivirus replication [22–24].

### HIV-1 regulates host FASN

**Fig. 2:**
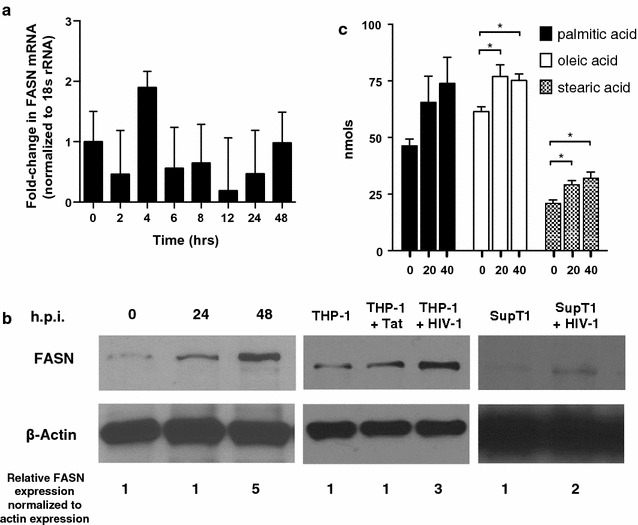
HIV-1 infection of TZM-bl cells does not alter FASN mRNA levels but it does increase FASN protein and fatty acid levels. **a** FASN mRNA levels, normalized to 18S rRNA levels, following infection with HIV-1_NL43_ for the indicated number of hours post infection (h.p.i). Normalized mRNA levels at 4 h post infection are not significantly different than normalized mRNA levels at time 0, p = 0.2, Student’s *t* test. Data are representative of two independent experiments. **b** Western blot analysis of FASN and actin protein levels in TZM-bl cell lysates [left], THP-1 lysates following pcTAT transfection or HIV-1 infection [middle], or SupT1 lysates [right] with or without HIV-1 infection. ImageJ software was used to calculate relative FASN expression normalized to actin expression. **c** Fatty acid quantification by gas chromatography was performed on extracts of TZM-bl cells following 48 h of infection with 0, 20, or 40 ng p24/mL of HIV-1. The data presented are mean values (± SD) from four independent experiments. * indicates p < 0.05 (Student’s *t* test). h.p.i = hours post infection

To validate the purinome-capture of FASN, we measured FASN expression in TZM-bl cells 24 and 48 h post HIV-1 infection. FASN mRNA levels, normalized to 18S rRNA, did not change following HIV-1 infection, suggesting FASN regulation in TZM-bl cells occurs post-transcriptionally (Fig. 2a). Western blotting with a FASN-specific antibody confirmed that HIV-1 infection increases FASN levels twofold to fivefold, as early as 24 h post infection; similar increases in FASN protein levels were also observed following HIV-1 infection of SupT1 and THP-1 cells (Fig. 2b). To determine if HIV-infection regulates FASN activity, we quantified intracellular fatty acid (FA) levels in TZM-bl cells with or with HIV-1. Our results show that 48 h post infection, HIV-1 increased intracellular palmitic, oleic, and stearic acid levels (Fig. 2c). Because FA extractions were performed from an equivalent number of cells, it is unlikely that increased FA levels were due to differences in cellular biomass.

**Fig. 3:**
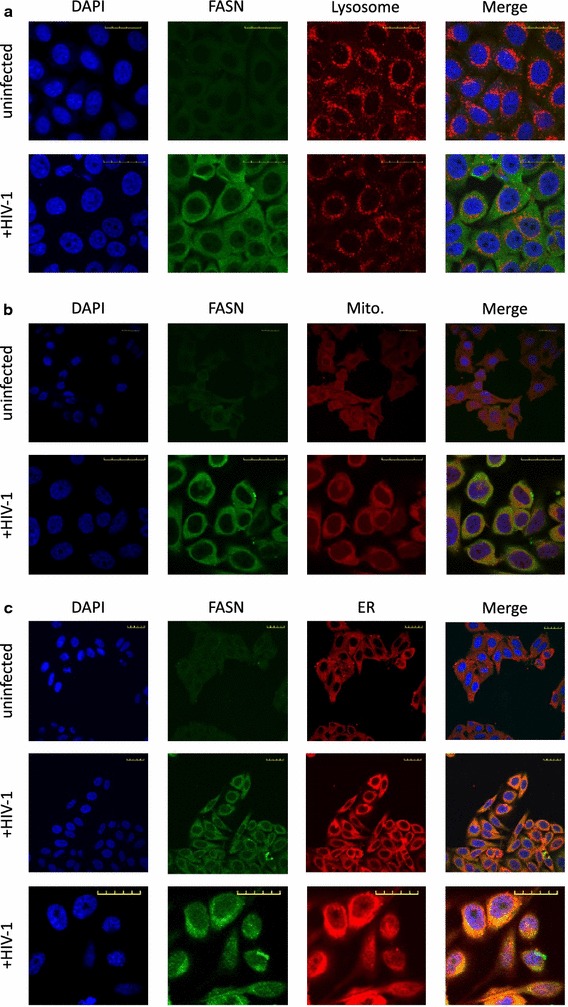
Incubation of TZM-bl cells with HIV-1 increases intensity of FASN staining but does not change FASN subcellular localization. In all panels, FASN is labeled green and the nucleus is colored blue (DAPI). Red color denotes **a** lysosome (CD63), **b** mitochondria [Mito] (Mitotracker), **c** endoplasmic reticulum [ER] (calreticulin). Yellow scale bar equals 20 µm. Data are representative of two independent experiments

FASN is a 272 kDa, multifunctional, cytosolic enzyme that uses NADPH to condense acetyl-CoA and malonyl-CoA into palmitate [25]. It has been shown that viral infections can change subcellular localization of FASN; for example, Dengue [22] infection causes FASN to relocalize to a perinuclear space, and Vaccinia virus infection relocalizes FASN to the mitochondria [26]. To determine if HIV-1 infection also causes FASN relocalization, we used immunofluorescence to monitor FASN distribution in HIV-1 infected TZM-bl cells. Although the intensity of FASN staining increased following HIV-1 infection, redistribution of FASN to a perinuclear space, lysosomes (Fig. 3a), mitochondria (Fig. 3b), or the endoplasmic reticulum (Fig. 3c) was not observed. Thus, similar to HCV [27], HIV-1 infection does not cause intracellular FASN redistribution.

### Fasnall is a novel FASN inhibitor that reduces HIV-1 replication

**Fig. 4:**
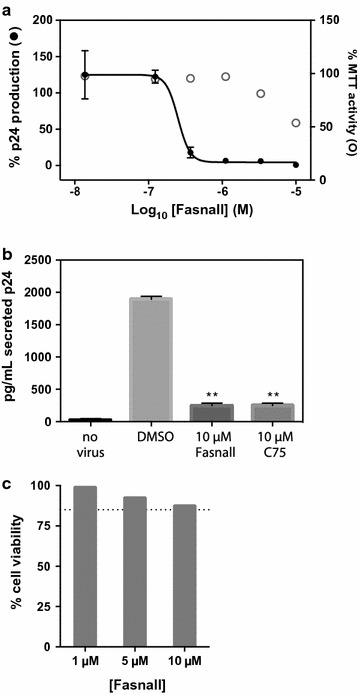
Fasnall inhibits HIV-1 replication. **a** Extracellular p24 levels in TZM-bl cells 48 h post infection (black dots ± SD, n = 3, black line), without significant effects on TZM-bl cell viability (i.e. mean MTT-activity; open circles, n = 3) **b** Fasnall and C75, each at 10 µM, significantly reduce HIV-1 replication in primary PBMC, as measured by p24 production. The data presented are mean values (± SD) from three independent experiments. ** indicates p < 0.0001, treatment versus DMSO-treated control (Students *t* test), **c** PBMC viability after treatment with Fasnall as measured by propidium iodide staining (dotted line drawn at 85%)

We recently reported the discovery of a thiophenopyrimidine molecule—Fasnall—that potently and selectively inhibits FASN activity in vitro and also demonstrates anti-tumor activity in vivo [15]. To determine if Fasnall blocks HIV-1 replication, we infected TZM-bl with HIV-1 and 48 h post infection measured extracellular p24 levels as surrogate measure of HIV-1 replication. In this model, Fasnall potently inhibited HIV-1 p24 production with an EC_50_ of 213 nM (95% CI 93–487 nM) and an estimated cellular toxicity (TC_50_) of 10 µM (Fig. 4a), resulting in an antiviral index (TC_50_/EC_50_) of 47. To determine if Fasnall blocked HIV-1 in activated T-cells, we measured p24 production from HIV-1 infected primary PBMCs in the presence or absence of 10 µM Fasnall. In this physiological relevant model of HIV-1 replication, Fasnall reduced HIV-1 p24 production approximately tenfold (Fig. 4b), with minimal effects on cell viability (Fig. 4c). Moreover, when PBMCs were treated with C75, a commercially available FASN inhibitor, similar reductions in extracellular p24 levels were observed (Fig. 4b). Thus, FASN activity is required for efficient HIV-1 replication in primary PBMCs.

### FASN activity modulates the late stages of HIV-1 replication

**Fig. 5:**
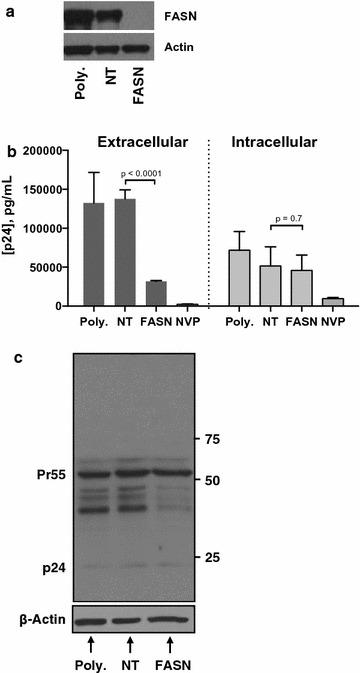
FASN knockdown reduces HIV-1 particle production without affecting intracellular Gag production. **a** FASN immunoblot confirms reduction in endogenous FASN levels in TZM-bl cells. **b** Extracellular (left) and intracellular (right) p24 levels following siRNA-mediated FASN knockdown in TZM-bl cells infected with HIV-1 for 48 h. Bars represent mean p24 ± SD, quantified using a commercial ELISA, n = 3, p-values generated with student’s *t* test. **c** anti-Gag western blot of lysates from HIV-infected cells with or without FASN knockdown. Molecular weight markers (kD) indicated in right margin. Poly = polymer (transfection) control, NT = nontargeting siRNA (siRNA control), FASN = FASN-targeted siRNAs, NVP = 0.4 µM nevirapine

To further confirm that the inhibitory effect of Fasnall and C75 was mediated by FASN, we transfected TZM-bl cells with either FASN-specific or non-targeting siRNAs (abbreviated *NT*). Following siRNA mediated FASN knockdown (Fig. 5a), cells were HIV-infected for 48 h, and extracellular p24 levels were again measured. Similar to the Fasnall results, siRNA-mediated FASN reduction reduced the levels of HIV-1 p24 released into the culture supernatant by 77%, compared to non-targeting siRNA control cells (Fig. 5b, left). Despite this decrease in culture supernatant p24 levels, siRNA-mediated FASN knockdown did not significantly reduce intracellular p24 levels, measured by ELISA (Fig. 5b, right), suggesting that HIV-1 replication uses FASN activity during a late step in HIV-1 replication (e.g. protein trafficking, virion assembly, or virion release from the cell). Anti-Gag western blot of HIV-infected, FASN-knockdown cells indicates similar levels of intracellular p55 and p24 (Fig. 5c).

**Fig. 6:**
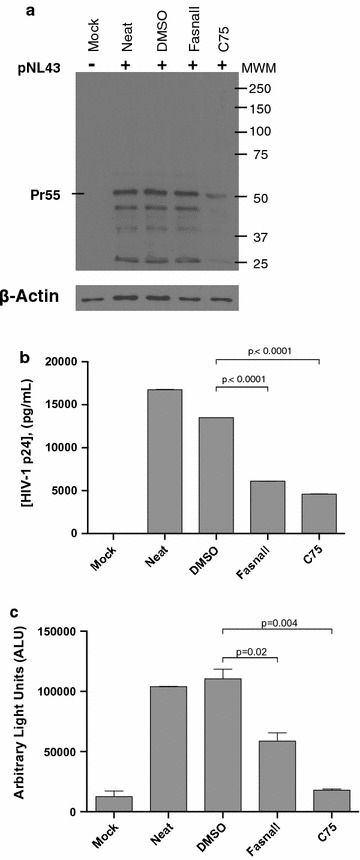
FASN inhibition disrupts a late step in the HIV-1 replication cycle. **a** TZM-bl cells were transfected with pNL4-3 provirus plasmid for 48 h in the presence or absence of 10 µM Fasnall or 10 µM C75 or DMSO (0.01%). Intracellular expression of HIV-1 proteins was monitored by HIVIG-western blot, and β-actin was used as loading control. **b** Supernatant associated virion production was monitored using a p24 ELISA. **c** 48 h post-transfection, cell culture supernatants were removed and incubated with fresh TZM-bl (indicator) cells for an additional 48 h. Values expressed as the mean ± standard deviation, and are representative of three independent experiments. p-values were generated with student’s *t* test. Molecular weight markers (kD) indicated in right margin

To test further the hypothesis that HIV-1 replication requires FASN activity during the late stages of viral replication, we transfected TZM-bl or SupT1 (data not shown) cells with a HIV-1 provirus (pNL43) in the presence or absence of Fasnall or C75. Similar to siRNA-based FASN knockdown, Fasnall-based inhibition of FASN did not reduce intracellular Gag levels (Fig. 6a) but did significantly reduce HIV-1 p24 particle deposition into culture medium, as measured by p24 production (Fig. 6b). Fasnall and C75 similarly reduced the number of infectious HIV-1 particles (Fig. 6c). Thus, FASN inhibition reduces nascent HIV-1 virion production without reducing HIV-1 protein synthesis.

## Discussion

Here we show that HIV-1 infection increases FASN expression, and using both siRNA and pharmacological tools, we show that FASN inhibition blocks a late stage of HIV-1 replication. We also report that Fasnall, a next-generation FASN inhibitor with in vivo antitumor activity, has potent anti-HIV activity in both cell-culture and PBMC-based models of HIV-1 replication. From these findings, we hypothesize that FASN is a host-dependency factor.

Several siRNA-based studies of HIV-1 host dependency factors have been published (reviewed in [7]), none of which reported FASN as a host dependency factor. Many enveloped viruses, including cytomegalovirus (CMV), DENV, Epstein-Barr virus, HCV, HBV, influenza virus, Respiratory syncytial virus (RSV), and West Nile Virus, also require host FASN activity to replicate efficiently [22–24, 28–30]. Building on these results, we now show that similar to many other enveloped viruses, efficient HIV-1 replication requires host FASN activity.

FASN expression is highly regulated in cells and can change ten-fold (or more) in response to physiological stresses such a starvation, lactation or pathological states [25]. FASN up-regulation has been observed in breast cancer, melanoma, and hepatocellular carcinoma [31]. Our in vitro results show that HIV-1 infection increases intracellular FASN levels in both TZM-bl and SupT1 cells. These results complement findings from others that showed HIV-1 infection increased FASN levels in both CEMx174 [32] and RH9 T-cells [33]. Similar changes in FASN levels have also been observed in people living with HIV-1; specifically, in a study of 191 people living with HIV-1, Aragones et al. [34] showed that people living with HIV-1 not taking antiretroviral therapy (ART) had elevated serum FASN levels compared to both HIV-negative people and people living with HIV-1 on ART. Thus, our results are consistent with previous in vitro and in vivo studies that correlated HIV-1 infection with increased FASN levels.

**Fig. 7:**
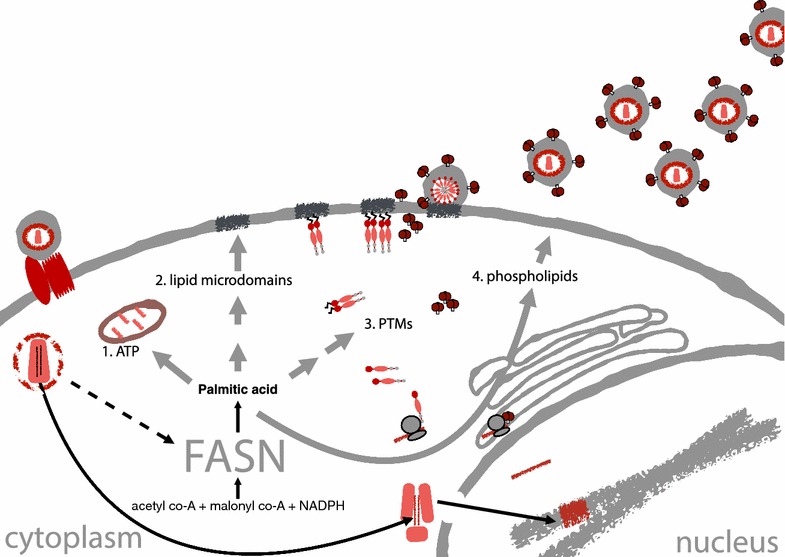
Potential mechanisms linking FASN activity to HIV-1 replication. (1) Provision of fatty acids used for ATP production and energy homeostasis, (2) creation of lipid micro domains (rafts) favoring HIV-1 budding, (3) generation of fatty-acyl adducts (e.g. palmitate or myristate) for post-translational modification (PTM) of Env, Gag, Nef, or host proteins, (4) homeostatic replenishment of membrane lipids lost during viral budding

FASN catalyzes the complete synthesis of palmitate from acetyl-CoA and malonyl-CoA into long-chain saturated FAs. FASN is a multifunctional enzyme that synthesizes FA chains two-carbons at a time, each donated from malonyl-CoA. The final product, palmitic acid (16:0) is then released, where it can be metabolized further by β-oxidation, into myristic acid (14:0), or other long chain FAs [25]. Long chain FAs are essential components of lipid bilayers, store energy liberated by β-oxidation, and FAs can be covalently attached to proteins as a means to control protein subcellular localization [35]. The results presented here show that FASN activity is required for efficient HIV-replication, but it remains unclear how de novo synthesized FA are used by HIV-1. Potential mechanisms (Fig. 7) include the following: (1) provision of FA’s used for ATP production and energy homeostasis, (2) creation of lipid micro domains associated with HIV-1 budding, (3) generation of fatty-acyl adducts (e.g. palmitate or myristate) for post-translational modification (PTM) of Env, Gag, Nef, or host proteins required for HIV-1 replication, or (4) replenishment of phospholipids to regenerate the lipid bilayer lost during viral budding. Available evidence suggests that FASN-dependent cancers likely regulate de novo FA biosynthesis to produce lipids for membrane synthesis and energy production (i.e. Fig. 7, mechanism 4) [36]. Vaccinia virus infection has been shown to cause FASN to relocalize to mitochondria, likely for energy homeostasis (i.e. Fig. 7, mechanism 1) [26]. Flaviviruses such as HCV and Dengue virus likely use FASN/FA to rearrange intracellular membranes to replicate their genomes on membranous webs, which is not represented in Fig. 7 [22, 23]. When FASN activity is inhibited in the context of HIV-1 infection, HIV-1 Gag is produced but viral particles are not released into the culture supernatant. Based on this observation, we expect hypotheses 2 and 3 offer the most plausible mechanisms by which HIV-1 leverages FASN activity: to generate FA to create cholesterol-rich lipid micro domains that promote viral budding [37–40], or to generate fatty-acyl adducts required for viral protein function [41–44]. Regarding hypothesis 3 (protein acylation), several HIV-1 proteins contain fatty-acyl modifications, including palmitoylation (Env, Nef), and myristoylation (Gag). Env has been shown to be palmitoylated, although the literature provides conflicting evidence regarding whether Env palmitoylation is required for Env function [45] or not [46]. Our results indicate that reduction of FASN activity affects HIV-1 replication post-translation, and this result is reminiscent of the phenotype observed when the N-terminal glycine of Gag is changed to an alanine [41, 47]. Specifically, it has been observed that Gag myristoylation is required for Gag–Gag multimerization [42], and mutation of the glycine at amino acid position two, which is required for matrix myristoylation, abolishes viral particle release [47]. Incubation of cells with alternative substrates of N-myristoyl transferases has been shown to block HIV-1 virus-like particle (VLP) production [43]. Thus, a plausible hypothesis states that HIV-1 stimulates de novo FA synthesis to produce myristate, which is required for Gag function.

FASN is considered a front-line drug target for the treatment of metabolic syndromes, cancer, and HCV [48–50], and data from this report suggest that FASN could be targeted in anti-HIV therapy. In adults, most normal tissues obtain FA exogenously from diet, and as a result, most cells have limited de novo FA biosynthesis and express FASN at very low levels [21]. Although studies in mice indicate that FASN is required for embryonic development [51], liver or macrophage-specific FASN knockout mice are viable [52, 53]. We recently identified a new FASN inhibitor (Fasnall) that showed potent antitumor activity in a MMTV-Neu model of breast cancer [15]. In that study, we reported that in mice, a 15 mg/mL intraperitoneal (IP) injection of Fasnall did not alter animal weight, and it also did not alter blood cell counts or markers of liver and kidney function [15]. At this same dose, we also observed that Fasnall achieved a peak plasma concentration of 27 µM, which is 150 times greater than the EC_95_ value of Fasnall against HIV-1 replication (in TZM-bl cells). As several enveloped viruses require FASN activity, we suggest that Fasnall could be a useful starting point in the development of a pan-antiviral therapy.

## Conclusions

Here we have shown that HIV-1 infection increases FASN expression. Using siRNA and small molecule FASN inhibitors, we have also shown that FASN activity contributes to HIV-1 replication during a late state in the viral replication cycle. Based on these observations, we hypothesize that FASN is a novel host dependency factor and that inhibition of FASN activity has the potential to be exploited as an antiretroviral strategy.

## Methods

### Reagents

All chemicals were obtained from Sigma-Aldrich (St Louis, MO, USA). Cells were obtained from ATCC (Manassas, Virginia, USA), except TZM-bl cells, which were obtained from the NIH AIDS Reagent Program (submitted by Dr. John C. Kappes, Dr. Xiaoyun Wu and Tranzyme Inc.). A plasmid containing a HIV-1 provirus (pNL4-3) was obtained through the NIH AIDS Reagent Program from Dr. Malcolm Martin. A plasmid containing HIV-1 Tat was also obtained through the Aids Reagent Program (pcTAT). HIV-1 concentration was determined with a commercial p24 ELISA assay kit (Zeptometix, Buffalo, NY). Anti-HIV-1 p24 Monoclonal antibodies (71-31 or cat# 530) and HIV-immunoglobulin (HIVIG; cat# 3957) were obtained from the AIDS Reagent Repository.

### Purinome capture

ATP Sepharose was synthesized as described [54]. HIV-1_NL4-3_ was produced in 293T cells according to standard protocols [55]. TZM-bl cells were lysed at 4 °C in lysis buffer (20 mM hepes pH 7.4, 1× complete protease inhibitors without EDTA (Roche, Indianapolis, IA), 120 mM NaCl, 20 mM MgCl_2_, 1 mM DTT, 0.1% NP-40), centrifuged at 16,000×*g* for 10 min at 4 °C, and the supernatant was loaded onto 100 µL ATP Sepharose. ATP-Sepharose was incubated with cell lysate for 1 h at 4 °C, washed 3× with low salt buffer (50 mM hepes pH 7.4, 120 mM NaCl, 20 mM MgCl_2_, 1 mM DTT), washed 2× with high salt buffer (low salt buffer with 300 mM NaCl [final]), then washed 2× with low salt buffer. Proteins were competed off the resin with 25 mM ATP dissolved in low salt buffer, dialyzed to remove ATP, mixed with Laemmli sample buffer, and visualized by 1-D SDS PAGE. Gels were fixed and silver stained according to published protocols [17]. Individual proteins were manually excised from the gel, washed, dehydrated, and digested in porcine trypsin (Promega, Madison, WI), as described [56]. Peptides were spotted on a MALDI plate (matrix: alpha-cyano-4-hydroxycinnamic acid (Aldrich Chemical Co. Milwaukee, WI) in a saturated solution of acetonitrile:25 mM aqueous ammonium citrate:trifuoroacetic acid (1:1:0.02, *v/v/v*). MALDI-MS/MS data were acquired using the AB Sciex 5800 TOF/TOF Mass Spectrometer (AB Sciex, Framingham, MA). Peptide mass fingerprint and peptide sequence data were resolved by the SPROT(UNIPROT) and NCBI databases using the Mascot search engine.

### FASN visualization

Equal amounts of cellular protein from TZM-bl cells with or without HIV-1 were separated on an 8% SDS-PAGE gel, transferred to nitrocellulose, immune-blotted with anti-FASN (Abcam, Cambridge, MA), then anti-rabbit secondary Ab (Abcam, Cambridge, MA), and visualized with ECL detection reagent (GE Biosciences). To verify equal protein loading, the membrane was stripped and probed with anti-actin (Cell Signaling, Danvers, MA). Protein levels were quantified using ImageJ software (https://imagej.nih.gov). For immuno-fluorescence experiments, 1 × 10^5^ TZM-bl cells were plated on sterilized coverslips, infected with 10 ng (p24)/mL HIV-1_NL4-3_ for 48 h, washed twice with PBS, fixed with 4% paraformaldehyde, washed thrice with PBS, permeabilized with chilled methanol, washed thrice with PBS, blocked with PBS + 1% BSA, incubated with anti-FASN primary Ab (Abcam, Cambridge, MA), and visualized with Alexa fluor-488-conjugated anti rabbit IgG (Life Technologies, New York, NY). Lysosomes were visualized using CD63 Ab ]Developmental studies hybridoma bank (DSHB), University of Iowa]; endoplasmic reticulum was stained with calreticulin Ab (DSHB, University of Iowa), and imaged with fluorescent secondary Ab (Alexa Fluor 594-anti mouse IgG, Life Technologies, New York, NY). Mitochondria were stained with mitotracker-red (Life Technologies, New York, NY). Coverslips were mounted onto fixed cells using Prolong Gold DAPI mounting medium (ThermoFisher, Waltham, MA) and observed on a FLUOVIEW Olympus microscope. Related images were collected for equivalent exposure times.

### Real time polymerase chain reaction (RT-PCR)

TZM-bl cells infected with HIV-1_NL4-3_ at 10 ng (p24)/mL were collected at intervals over 48 h of infection and total RNA was isolated using Qiagen RNeasy kit. Synthesis of cDNA was performed using oligo dT primer and Superscript III Reverse Transcriptase (Invitrogen, Carlsbad, CA). Real time PCR using SYBR green kit was performed according to manufacturer’s instructions (BioRad, Hercules, CA). The FASN primers (sense, 5′-CCCACCTACGTACTGGCCTA-3′; antisense, 5′-CTTGGCCTTGGGTGTGTACT-3′) were used to synthesize the PCR products. The 18s ribosomal RNA subunit primers (sense, 5′-CAGCCACCCGAGATTGAGCA-3′; antisense, 5′-TAGTAGCGACGGGCGGTGTG-3′) were used as controls to normalize FASN samples. PCR was run for 40 cycles, with 1 cycle consisting of 30 s at 95 °C, 30 s at 55 °C, and 30 s at 72 °C.

### SiRNA knockdown of FASN

ON-TARGET plus SMART pool of four siRNAs targeted against human FASN (FASN) (L-003954-00-0005) and ON-TARGET plus non-targeting (NT) control siRNA (D-001810-01-05) were purchased from Dharmacon (Thermo Fisher Scientific, Waltham, MA). TZM-bl cells were transfected either with 200 nM FASN-targeting siRNA or 200 nM NT siRNA using Trans-IT transfection reagent (Mirus Bio LLC, Madison, WI). After 48 h, cells were infected with 10 ng (p24)/mL HIV-1_NL4-3_, incubated for 24 h, medium was replenished with fresh medium, and incubated for additional 24 h. Supernatants were collected for HIV-1 p24 ELISA and cells were washed with PBS and saved for Western blotting.

### Fatty acid analysis and quantification

TZM-bl cells were infected with 0, 20, or 40 ng (p24)/mL HIV-1_NL4-3_ and total fatty acids were extracted from an equivalent number of cells using a modified version of the Bligh and Dyer protocol [57]. This consisted of sequential extractions with chloroform:methanol (2:1, 1:1 and 1:2, *v/v*), and chloroform:methanol:water (10:10:3, *v/v/v*). Fatty acid methyl esters were generated by methanolysis with 3 N methanolic-HCl (85 °C overnight) followed by trimethylsilylation with Tri-Sil reagent (Thermo Scientific, Waltham, MA). Heptadecanoic acid (17:0) was used as internal standard. Samples were dissolved in hexane prior to injection on a Thermo Scientific Trace GC–MS ULTRA/DSQII with a Rtx-5MS column (30 m × 0.25 mm internal diameter, 0.25 μm film thickness, Restek Corporation, Bellefonte, PA). Instrument settings included an internal temperature of 150 °C for 3 min, increasing to 200 °C at 2 °C/min and to 250 °C at 40 °C/min holding for 4 min.

### TZM-bl infection assays

HIV-1_NL4-3_ was added to 3 × 10^4^ TZM-bl cells at 10 ng (p24)/mL equivalents in the presence of 7.5 μg/mL DEAE dextran. Fasnall or C75 were serially diluted in DMSO, and all assays had a final DMSO concentration of ≤ 1%. HIV-infected cells were incubated at 37 °C in 5% CO_2_ for 24 h, washed with PBS, fresh medium was added, and cell were incubated for another 24 h. HIV-1 replication was assessed by quantitative p24 ELISA. Nevirapine (positive control) was obtained from the AIDS Reagent Repository.

### Primary cell infection

PBMCs were isolated by Ficoll-Paque centrifugation, stimulated in complete RPMI-1640 medium (Gibco, Carlsbad, CA) containing 10% FBS, 100 µg/mL penicillin/streptomycin and supplemented with 5 µg/mL phytohemagglutinin (PHA; Gibco) for 48 h, and maintained thereafter in complete RPMI-1640 medium supplemented with 20 U/mL of interleukin-2 (Gibco). PBMCs were seeded in 24-well plate (2 × 10^5^ cells/well) and triplicate wells were treated with indicated concentrations of C75, FASNALL, or with DMSO, and subsequently infected with 10 ng (p24)/mL equivalents of HIV-1_NL4-3_. Cells were washed 24 h post infection, and supernatants were collected and p24 content analyzed by quantitative ELISA (Zeptometrix, Buffalo, NY). PBMC viability was assessed by flow cytometry using propidium iodine (PI) exclusion (BD Pharmingen).
